# Inbuilt Potential of YEM Medium and Its Constituents to Generate Ag/Ag_2_O Nanoparticles

**DOI:** 10.1371/journal.pone.0061750

**Published:** 2013-04-23

**Authors:** G. Yamal, P. Sharmila, K. S. Rao, P. Pardha-Saradhi

**Affiliations:** 1 Department of Environmental Studies, University of Delhi, Delhi, India; 2 Department of Botany, University of Delhi, Delhi, India; Dowling College, United States of America

## Abstract

We discovered that Yeast Extract Mannitol (YEM) medium possessed immense potential to generate silver nanoparticles from AgNO_3_ upon autoclaving, which was evident from (i) alteration in color of the medium; (ii) peak at ∼410 nm in UV-Vis spectrum due to surface plasmon resonance specific to silver nanoparticles; and (iii) TEM investigations. TEM coupled with EDX confirmed that distinct nanoparticles were composed of silver. Yeast extract and mannitol were key components of YEM medium responsible for the formation of nanoparticles. PXRD analysis indicated crystalline geometry and Ag/Ag_2_O phases in nanoparticles generated with YEM medium, yeast extract and mannitol. Our investigations also revealed that both mannitol and yeast extract possessed potential to convert ∼80% of silver ions in 0.5 mM AgNO_3_ to nanoparticles, on autoclaving for 30 min at 121°C under a pressure of 1.06 kg/cm^2^. Addition of filter sterilized AgNO_3_ under ambient conditions to pre-autoclaved YEM medium and yeast extract brought about color change due to the formation of silver nanoparticles, but required prolonged duration. In general, even after 72 h intensity of color was significantly less than that recorded following autoclaving. Silver nanoparticles formed at room temperature were more heterogeneous compared to that obtained upon autoclaving. In summary, our findings demonstrated that (i) YEM medium and its constituents promote synthesis of silver nanoparticles; and (ii) autoclaving enhances rapid synthesis of silver nanoparticles by YEM medium, yeast extract and mannitol.

## Introduction

Nanotechnology is one of the most advancing areas of research in modern material science. Nanoparticles exhibit completely new or improved physical (in particular optical, electronic, magnetic) and chemical properties. Silver (Ag) nanoparticles possess strong antimicrobial effects, making them suitable for use in medical devices such as catheters, wound dressings, skin donation, prostheses, contraceptives, in daily commodities such as shampoos, soaps, toys, paints, textile, shoe insoles, washing machines etc. [1]–[3]. Nanoparticles of silver also show the property of surface plasmon resonance, rendering them to be used in diagnostics and sensing applications [3]–[6].

Various physical, chemical and physico-chemical approaches such as the use of laser ablation, mechanical milling, inert gas condensation, thermal irradiation, laser irradiation, chemical reduction, photochemical reduction and electrochemical techniques have been demonstrated to generate metal nanoparticles [7]–[11]. But, the majority of these methods are expensive and/or the byproducts are potentially dangerous to the environment [11]. Sodium borohydrate and sodium citrate methods originally discovered by Brust et al. [12] and Turkevich et al. [13], respectively remain the most popularly used reductants for generation of metal nanoparticles. Owing to the wide applicability and increased commercial demand, the desire to generate nanoparticles in most economically viable and environmentally friendly ways has gained impetus.

During an attempt to assess the potential of a symbiotic bacterium *Rhizobium* sp. to synthesize silver nanoparticles [using silver nitrate (AgNO_3_)], we noted the change in color of yeast extract mannitol (YEM) medium supplemented with AgNO_3_ from pale yellow to brown up on autoclaving. Such a change in color of medium supplemented with AgNO_3_ is often associated with the formation of silver nanoparticles. This prompted us to carry out investigations for identifying the component(s) of YEM medium responsible for generation of silver nanoparticles.

In this communication, we report for the first time that the YEM medium and two of its components namely mannitol and yeast extract have potential to synthesize silver nanoparticles from Ag^+^ under aqueous conditions. Our investigations revealed that autoclaving promotes rapid synthesis of stable, well dispersed silver nanoparticles in aqueous phase and the same has been compared with the production of silver nanoparticles under ambient conditions.

## Materials and Methods

Mannitol, Yeast Extract, K_2_HPO_4_, MgSO_4_.7H_2_O and NaCl were of Himedia (India) make. AgNO_3_ was obtained from Merck. All the chemicals and reagents used were of Analytical grade, and double distilled water was used for all the investigations. YEM broth composed of 53 mM Mannitol, 2.8 mM K_2_HPO_4_, 0.81 mM MgSO_4_.7H_2_O, 1.7 mM NaCl and 1 g/L yeast extract [14]. For evaluating the interaction of Ag^+^ with YEM medium, the later was supplemented with 0, 0.1, 0.25 and 0.5 mM AgNO_3_. In order to establish the component of YEM medium that resulted in the synthesis of silver nanoparticles upon autoclaving, individual components namely, Mannitol, Yeast Extract, K_2_HPO_4_, MgSO_4_.7H_2_O and NaCl were used (as per the concentrations in YEM medium) with 0.25 mM AgNO_3_. The pH in all cases was adjusted to 6.8. The media were dispensed into test tubes and placed in ∼75°C pre-heated vertical autoclave (Yorco, Delhi, India) and autoclaved for 30 min at 121°C under a pressure of 1.06 kg/cm^2^ (∼10 min was required for attaining a temperature of 121°C). About 20–30 min was taken for bringing down the autoclave to atmospheric pressure and temperature. After initial screening, the potential of mannitol and yeast extract to generate silver nanoparticles was evaluated with 0, 0.1, 0.25 and 0.5 mM AgNO_3_.

In order to test, if silver nanoparticles can be generated at room temperature, YEM medium, yeast extract and mannitol were autoclaved and after cooling different levels of filter sterilized AgNO_3_ were added under sterile conditions and incubated for different time intervals under ambient conditions.

### Characterization of Silver Nanoparticles

The presence of silver nanoparticles in the resultant colloidal solution was established through UV-Vis spectroscopy, transmission electron microscopy (TEM) and powder X-ray diffraction (PXRD) analysis.

The production of silver nanoparticles was initially confirmed by the UV-Vis spectrum of the resultant colored colloidal solutions. The spectra of the media were recorded using Analytikjena Specord 200 at a resolution of 10 nm and a scan rate of 20 nm/sec, to ascertain the presence of peaks arising due to surface plasmon resonance specific to silver nanoparticles.

The colored colloidal solutions obtained on autoclaving AgNO_3_ in combination with YEM broth, yeast extract or mannitol were centrifuged at 15,000×g for 30 min at 25°C. The resultant pellet formed was suspended in desired amount of water and used for TEM and PXRD studies.

For TEM investigations the suspended pellet was subjected to sonication for 30 min. 10 µl of the sonicated solution was drop coated onto a 200 mesh copper TEM grid with an ultrathin continuous carbon film and allowed to dry in a desiccator. The grids were viewed in the transmission electron microscope (Technai G2 T30 U-TWIN) at a voltage of 300 kV. The hardware associated with the machine also allowed (i) energy dispersive X-ray (EDX) to measure the elemental composition and (ii) selected area electron diffraction (SAED) pattern to reveal the crystalline nature of nanoparticles.

For PXRD studies the pellet suspended in water, was drop coated on the silica surface, and dried in the desiccator. The PXRD pattern was collected using RIGAKU ROTAFLEX RAD-B using Cu target CuK(α) 1 radiation with tube voltage 40 kV and 60 mA in the 2θ (degree) range of 20–80°.

### Yield

For estimating the product yield, resultant colloidal solutions were centrifuged at 57438×*g* in Sigma 3K30 centrifuge to obtain supernatant and the pellet. Silver left in the supernatant was determined with GBC Scientific Equipment SensAA atomic absorption spectrophotometer (AAS) at 328.1 nm.

## Results

**Figure 1:**
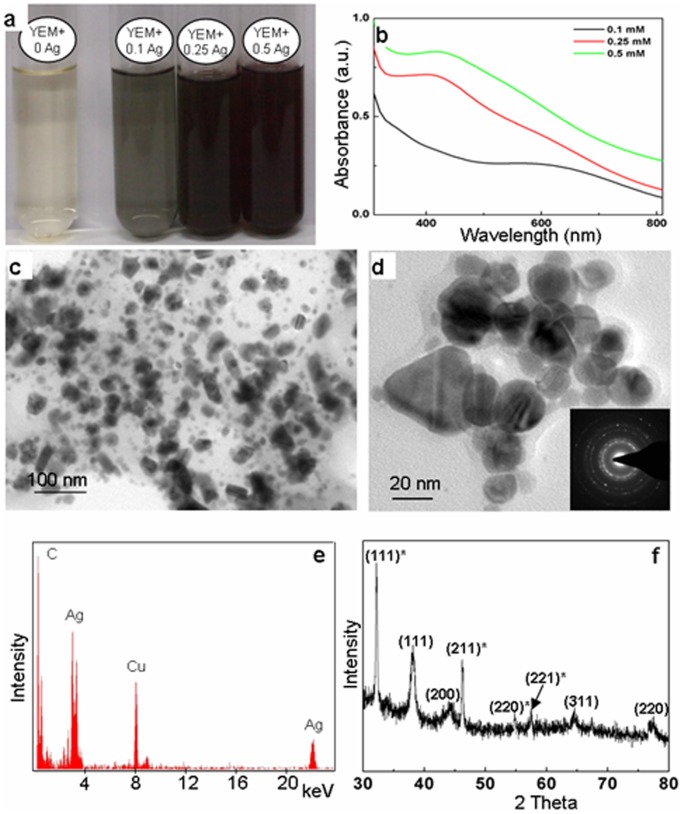
Evaluation of silver nanoparticles formed by autoclaving AgNO_3_ with Yeast Extract Mannitol (YEM) medium. **a**: Alteration in color of YEM medium autoclaved in presence of 0, 0.1, 0.25 and 0.5 mM AgNO_3_. **b**: UV-Vis absorption spectra of 0.1, 0.25 and 0.5 mM AgNO_3_ autoclaved with YEM medium showing the characteristic peaks for silver nanoparticles. **c & d**: TEM images of the silver nanoparticles. **Inset in d**: SAED pattern of nanoparticles. **e**: The EDX spectrum of nanoparticles showing characteristic peaks of Ag, indicating that the nanoparticles to be composed of silver. Other prominent peaks of C and Cu, noted in the figure arose from the carbon coated copper grids. **f**: The PXRD pattern of nanoparticles.

The color of the YEM broth in combination with AgNO_3_ changed from pale yellow to brown on autoclaving (Figure 1a). However, YEM broth and AgNO_3_ solutions retained their original color when autoclaved individually at 121°C under a pressure of 1.06 kg/cm^2^ for 30 min. It is well known, that colorless AgNO_3_ solutions turn brown due to the formation of silver nanoparticles. Accordingly, distinct peaks were seen in UV-Vis spectra at ∼410 nm (Figure 1b), which are well documented to be due to the surface plasmon resonance in silver nanoparticles [15]. As is evident from figure 1b, the intensity of peaks increased with increase in concentration of AgNO_3_.

**Figure 2:**
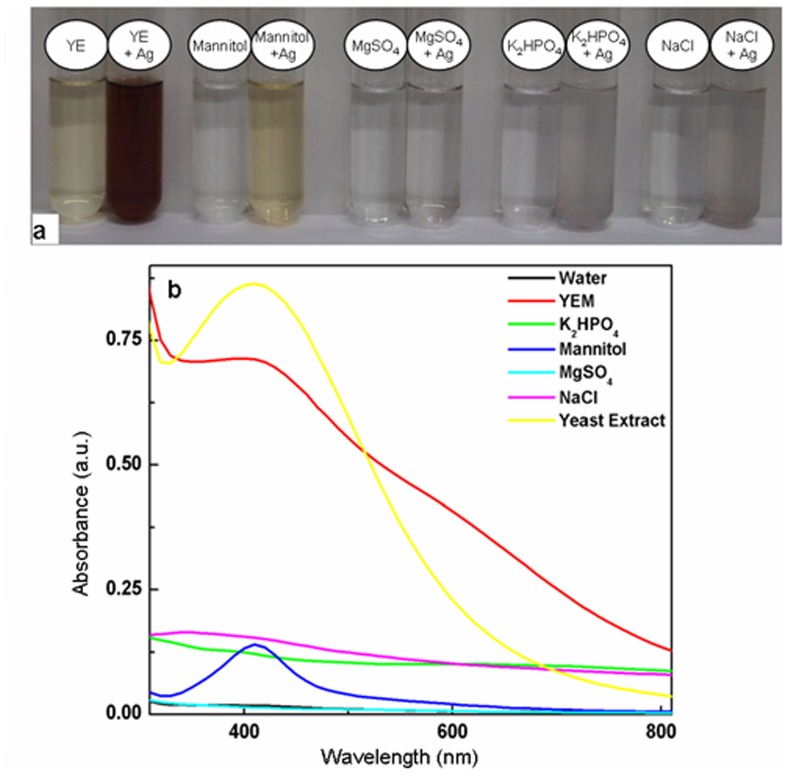
Evaluation of contribution of YEM medium components for silver nanoparticles generation upon autoclaving with AgNO_3_. **a**: Alteration in color of 0.25 mM AgNO_3_ upon autoclaving with different components of YEM medium namely, yeast extract (YE), mannitol, MgSO4, NaCl and K2HPO4 medium individually. **b**: UV-Vis absorption spectra of 0.25 mM AgNO_3_ after autoclaving with YEM medium, yeast extract, mannitol, MgSO4, NaCl and K2HPO4.

YEM is a complex medium with mannitol, yeast extract, K_2_HPO_4_, MgSO_4_.7H_2_O and NaCl as its constituents [14], therefore it was necessary to identify the key component(s) of YEM medium responsible for the formation of silver nanoparticles. Alteration in color of different components of YEM medium when used independently in combination with AgNO_3_ is depicted in figure 2a. As is evident from figure 2a, color of 0.25 mM AgNO_3_ turned brown in the presence of mannitol or yeast extract, but remained unaltered in combination with other components. Accordingly, the UV-Vis spectra of colored colloidal solution formed with AgNO_3_ in combination with yeast extract and mannitol showed absorption peak at ∼410 nm, while no peaks were seen with K_2_HPO_4_, NaCl and MgSO_4_.7H_2_O (Figure 2b).

**Figure 3:**
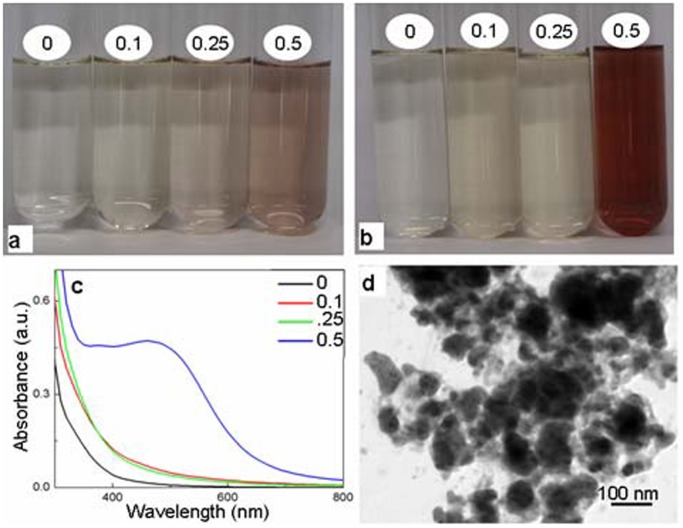
Evaluation of silver nanoparticles formed by incubating filter-sterilized AgNO_3_ with pre-autoclaved YEM medium under ambient conditions. **a–b**: Alteration in color of YEM medium incubated in presence of 0, 0.1, 0.25 and 0.5 mM AgNO_3_ after ∼8 h (a) and 72 h (b). **c**: UV-Vis absorption spectra of 0, 0.1, 0.25 and 0.5 mM AgNO_3_ incubated with YEM medium for 72 h. **d**: TEM image of silver nanoparticles formed under ambient conditions.

**Figure 4:**
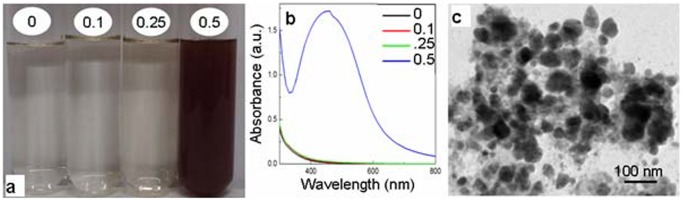
Evaluation of silver nanoparticles formed by incubating filter-sterilized AgNO_3_ with pre-autoclaved yeast extract (YE) under ambient conditions. **a**: Alteration in color of YE with 0, 0.1, 0.25 and 0.5 mM AgNO_3_ after 72 h. **b**: UV-Vis absorption spectra of YE incubated with 0, 0.1, 0.25 and 0.5 mM AgNO_3_ for 72 h. **c**: TEM images of silver nanoparticles formed under ambient conditions.

In order to evaluate if autoclaving is essential for formation of silver nanoparticles by YEM medium and its constituents, different levels of filter-sterilized AgNO_3_ were added to pre-autoclaved, cooled YEM medium, yeast extract and mannitol and incubated under ambient conditions. Incubation of pre-autoclaved YEM medium and yeast extract with filter sterilized AgNO_3_ under ambient conditions also lead to the color change and the synthesis of silver nanoparticles. However, the color changed only after a prolonged duration of incubation. As evident from figure (Figure 3a) pre-autoclaved YEM medium as well as yeast extract although initiated color change in a duration of ∼16 h, the intensity of color of these media/solutions remained lower than that noted through autoclaving even after 72 h of incubation (Figures 3b–4a). Pre-autoclaved mannitol incubated with filter-sterilized AgNO_3_ under ambient conditions showed only minor color change. It is important to appreciate that the total duration required for generation of silver nanoparticles from beginning till the end of autoclaving was ∼1 h. In general, the silver nanoparticle specific absorption peak was more broader with the solutions containing silver nanoparticles formed under ambient conditions compared to those formed following autoclaving. The TEM investigations revealed the presence of nanoparticles in the YEM medium (Figure 3d) and yeast extract (Figure 4c) incubated with AgNO_3_ for 72 h under ambient conditions. It is also evident from the TEM images that the silver nanoparticles formed under ambient conditions were highly heterogeneous and agglomerated. In light of these negative aspects of formation of silver nanoparticles by YEM medium and yeast extract under ambient conditions, further investigations for generation and detailed characterization were restricted to the nanoparticles generated via autoclaving.

**Figure 5:**
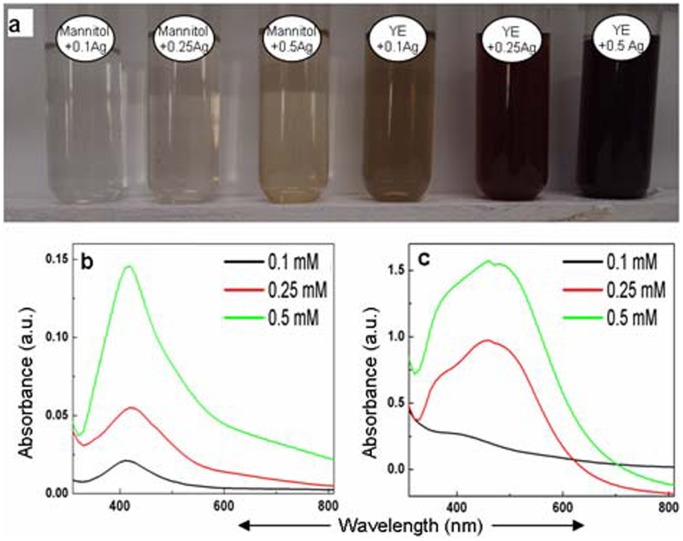
Evaluation of silver nanoparticles formed by autoclaving AgNO_3_ with yeast extract (YE) and mannitol. **a**: Alteration in color of 0.1, 0.25 and 0.5 mM AgNO_3_ autoclaved with 53 mM mannitol and 0.1% yeast extract. **b–c**: UV-Vis absorption spectra of 0.1, 0.25 and 0.5 mM AgNO_3_ after autoclaving with 53 mM mannitol and 0.1% yeast extract, respectively.

Figure 5a shows yeast extract and mannitol autoclaved with different levels of AgNO_3_. A distinct increase in the intensity of color was recorded with increasing concentration of AgNO_3_ (Figure 5a). Accordingly, intensity of absorption peak due to surface plasmon resonance of silver nanoparticles was intensified with increasing concentration of AgNO_3_, both in case of mannitol and yeast extract (Figures 5b–c). However, irrespective of the concentration of AgNO_3_, the intensity of the color of the solution and absorption peak due to silver nanoparticles was significantly higher with yeast extract compared to mannitol. The absorption peak obtained due to surface plasmon resonance in silver nanoparticles was broad in case of yeast extract and sharp in case of mannitol.

TEM analysis further established the presence of silver nanoparticles in YEM medium autoclaved with AgNO_3_. Figures 1c–d are the representative TEM images of silver nanoparticles formed in YEM medium supplemented with AgNO_3_. As is evident from these figures, the nanoparticles varied in size from 10–50 nm. The EDX pattern (Figure 1e) collected from these nanoparticles showed distinct peaks at 3.40 keV and 22 keV corresponding to Ag, while the peaks situated at binding energies of 8.06 and 1 keV correspond to Cu and C, respectively. The peaks of C and Cu arose due to their presence as an integral component of carbon coated copper grids.

The SAED pattern revealed the crystalline nature of silver nanoparticles (inset Figure 1d), which was further corroborated by the PXRD studies (Figure 1f). The PXRD pattern showed the Bragg reflections that can be assigned to (111), (200), (220) and (311) and matched with the standard diffraction pattern of Joint Committee on Powder Diffraction Standards (JCPDS) No. 04–0783, confirming the face-centered cubic (fcc) structure. The PXRD pattern also showed additional peaks at 2θ (degree) position of 32.31, 46.28, 54.83, 57.97, 67.95 which can be due to Ag_2_O cubic structure with Bragg reflections (111), (211), (220), (221) and (222).

**Figure 6:**
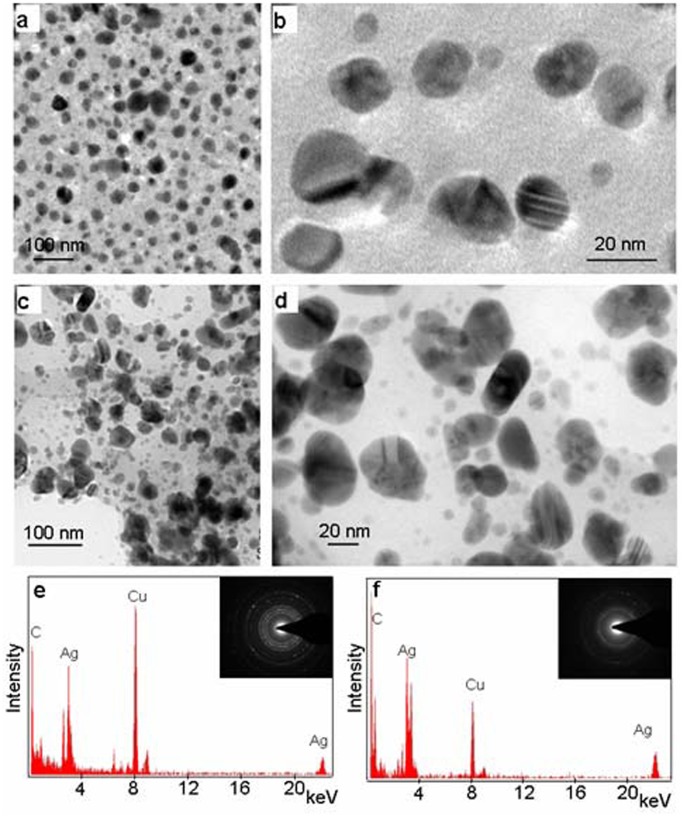
Characterization of silver nanoparticles formed by autoclaving AgNO_3_ with mannitol and yeast extract (YE). **a–b**: TEM images of silver nanoparticles synthesized with 53 mM Mannitol. **c–d**: TEM images of silver nanoparticles synthesized with 0.1% YE. **e–f**: EDX spectra of nanoparticles formed with mannitol and YE, respectively, showing the peaks for Ag, indicating nanoparticles to be composed of silver. Other prominent peaks of C and Cu, noted in the figure are due to the carbon coated copper grids. **Inset e–f**: SAED pattern of the silver nanoparticles formed with mannitol and YE, respectively, showing the crystalline nature of the nanoparticles.

TEM images of silver nanoparticles formed on autoclaving AgNO_3_ in combination with 53 mM (∼0.9%) mannitol or 0.1% yeast extract are shown in figures 6a–b and figures 6c–d, respectively. Silver nanoparticles formed in presence of mannitol were nearly spherical and varied in size range from 10–20 nm, while those formed with yeast extract varied in shape and were in the size range of 10–50 nm. The SAED pattern (insets in figures 6e–f) indicated the crystalline nature of these silver nanoparticles. The EDX spectra (Figures 6e–f) collected from these nanoparticles showed peaks for Ag, confirming that the nanoparticles were composed of silver.

**Figure 7:**
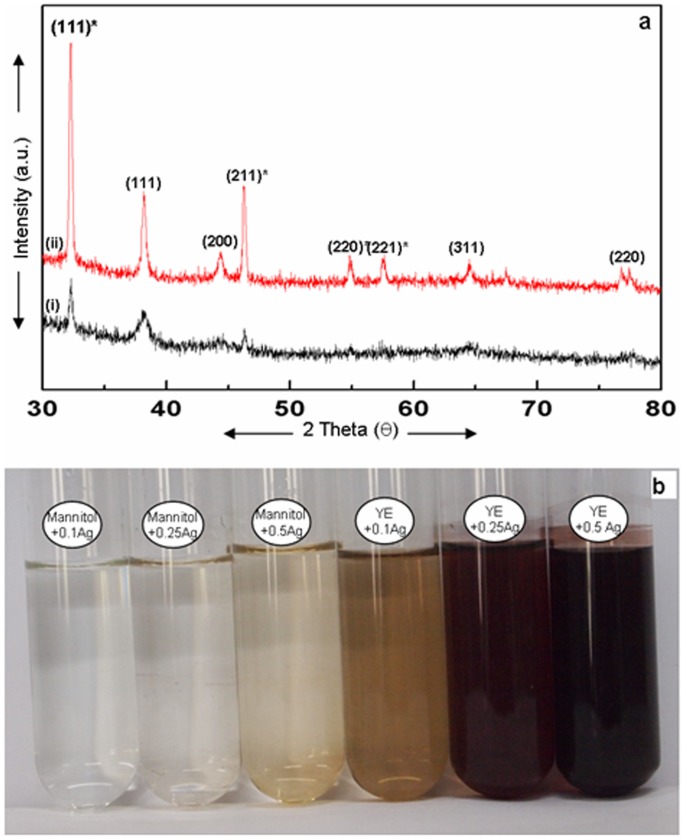
PXRD pattern and stability of silver nanoparticles formed upon autoclaving AgNO_3_ with mannitol and yeast extract (YE). **a**: PXRD pattern of the silver nanoparticles formed with (i) YE and (ii) mannitol showing bragg reflections characteristic of crystalline face-centred cubic structure of Ag () and cubic structure of Ag_2_O ()*. **b**: Ag nanoparticles formed from 0.1, 0.25 and 0.5 mM AgNO_3_ autoclaved independently with 53 mM mannitol and 0.1% YE after 1 month with no signs of agglomeration.

The PXRD pattern showed Bragg reflections that can be assigned to (111), (200), (220), (311) planes, and matched with JCPDS No. 04–0783. These results confirmed that silver nanoparticles synthesized with mannitol as well as yeast extract were crystalline with fcc structure. In a manner similar to YEM medium, the PXRD patterns of silver nanoparticles formed with mannitol and yeast extract (Figure 7a) also showed additional peaks, indicating biphasic nature of nanoparticles.

The AAS studies reveal that yeast extract as well as mannitol have equal potential to generate silver nanoparticles. Lower concentrations (viz. 0.1 and 0.25 mM) of AgNO_3_ autoclaved independently with yeast extract and mannitol did not show the presence of any silver ions in the supernatant, as they might have been converted into nanoparticles that pelleted down following centrifugation at 57438×g. However, 0.5 mM AgNO_3_ autoclaved independently with yeast extract and mannitol retained about 20% silver in ionic state.

The silver nanoparticles formed with mannitol as well as yeast extract were well dispersed and stable, and showed no signs of agglomeration even on storing for a period of over a month under ambient conditions (Figure 7b).

## Discussion

During present investigations, we observed alteration in color of YEM medium supplemented with AgNO_3_ from pale yellow to brown just upon autoclaving even without the inoculation of any microorganism. It has been established that colorless AgNO_3_ solution turns yellow to brown due to the formation of silver nanoparticles [16]. TEM investigations coupled with EDX studies confirmed the presence of silver nanoparticles in the YEM medium autoclaved with AgNO_3_ and accordingly this medium showed absorption peak at ∼410 nm, which is known to arise due to surface plasmon resonance in the silver nanoparticles [15]-[17]. In addition to the role of bacteria in the synthesis of silver nanoparticles demonstrated in earlier investigations, the present study also demonstrates the important ability of media components (YEM medium and two of its components viz. yeast extract and mannitol) to synthesize silver nanoparticles.

YEM is a complex medium constituted of yeast extract, mannitol, MgSO_4_, K_2_HPO_4_ and NaCl [14]. AgNO_3_ autoclaved independently with different components of YEM medium clearly indicated that two of its constituents namely, mannitol and yeast extract possessed potential to generate silver nanoparticles. As expected, silver nanoparticles generated with mannitol and yeast extract also showed absorption peaks at ∼410 nm due to their specific surface plasmon resonance. However, the absorption peak obtained with yeast extract was broader when compared to mannitol, indicating that the nanoparticles formed with the former were heterogeneous. The heterogeneity of silver nanoparticles formed by yeast extract was also evident from TEM images which not only showed variation in shape but also exhibited significant variation in size. Such a heterogeneous population of nanoparticles might have resulted due to the complex composition of yeast extract [18], [19].

Both yeast extract and mannitol possessed potential to convert cent percent of silver ions into nanoparticles when autoclaved with at concentrations of AgNO_3_ upto 0.25 mM. However, about 80% of silver ions were converted into nanoparticles with 0.5 mM AgNO_3_ within a duration of 1 h on autoclaving. The retention of 20% of silver ions in 0.5 mM AgNO_3_ indicated that the level of yeast extract and mannitol used was not sufficient for reducing 100% silver into nanoparticles. We believe that it is important to increase the level of mannitol or yeast extract depending on the concentration of AgNO_3_ used for obtaining optimal yield of silver nanoparticles, i.e. for converting cent percent of silver ions into nanoparticles.

During the course of present studies, experiments were also carried to assess, if silver nanoparticles can be generated under ambient conditions by adding and incubating filter sterilized AgNO_3_ to cooled, pre-autoclaved YEM medium, yeast extract and mannitol. This revealed that the former two possessed potential to alter the color and generate silver nanoparticles, but this alteration in color and the formation of silver nanoparticles under ambient conditions required prolonged duration. Infact, the intensity of the color of YEM medium as well as yeast extract incubated with AgNO_3_ under ambient conditions for a duration of 72 h was significantly less than that noted in a duration of 1 h through autoclaving. Further, TEM investigations revealed that silver nanoparticles formed under ambient conditions were more heterogenous when compared to those formed through autoclaving.

### Advantages of Autoclaving for Generation of Silver Nanoparticles

Addition of AgNO_3_ to pre-autoclaved cooled medium required prolonged duration for the generation of silver nanoparticles under ambient conditions and reasonable quantity of silver nanoparticles could be generated after ∼72 h. In contrast, autoclaving AgNO_3_ independently with YEM medium, yeast extract and mannitol promoted generation of large quantities of silver nanoparticles within a duration of 1 h. Further, the nanoparticles generated under ambient conditions were more heterogeneous when compared to those obtained through autoclaving.

The stability of nanoparticles depends on the medium, inter-particle forces and chemical reactivity, which affect aggregation, size and shape of the nanoparticles [20]. The production and stability of silver nanoparticles in aqueous phase remained a major challenge and it is important to develop novel strategies for their wide application. The silver nanoparticles produced by autoclaving during present investigations were well dispersed and retained stability even after a duration of more than a month (without showing any signs of agglomeration) in aqueous phase at room temperature.

For apt application in medicine and pharmaceutical industry it is important to generate silver nanoparticles under sterile conditions. We believe that autoclaving is a suitable approach for generation of metal nanoparticles under sterile conditions, as autoclaving is widely used for general sterilization purposes.

### Mechanism of Formation of Silver Nanoparticles by Yeast Extract

Yeast extract being autolysate of yeast cells is a cocktail of proteins/polypetides, amino acids, carbohydrates, vitamins etc. [18], [19] and few of these biomolecules must have played a vital role in the generation of silver nanoparticles. It is well established that various biomolecules such as peptides, amino acids, carbohydrates, vitamins etc. possess capacity to synthesize silver nanoparticles and the mechanism of synthesis of metal nanoparticles (from their ions) by different biomolecules vary significantly from each other as has been extensively elaborated by Duran et al. [21]. This accounts for the heterogeneity in the shape and size of nanoparticles obtained with yeast extract during present investigations. Some of the key groups of biomolecules in the cocktail of yeast extract namely (i) carboxyl group of glutamate and aspartate, and hydroxyl group of tyrosine, serine and threonine present individually or associated with polypeptides; and (ii) aldehyde, keto and hydroxyl groups associated with the carbohydrates, must have contributed significantly towards the reduction of Ag^+^ and formation and stabilization of silver nanoparticles. Similarly, vitamins such as riboflavin present in yeast extract could have also played an important role in generation of silver nanoparticles as has been reported earlier [22].

### Mechanism of Formation of Silver Nanoparticles by Mannitol

The nanoparticles formed with mannitol were relatively uniform in shape and size. In all likelihood mannitol might have generated silver nanoparticles through reduction of Ag^+^ to Ag^0^ following Fetizon oxidation mechanism which involves oxidation of a primary alcohol to an aldehyde group followed by further oxidation to form lactone/ketone [23], [24]. Ag^0^ can further nucleate to generate silver nanoparticles.

For effective commercial endeavors, it is important to generate nanoparticles of uniform size and shape. It is equally important to ensure that the molecules/agent used for generating nanoparticles are completely safe and compatible with living systems and must be eco-friendly. Unlike yeast extract, which is a complex mixture of several biomolecules, mannitol has been well established to be a compatible solute, which has potential to protect living systems against various stresses [25]–[29].

The generation of stable nanoparticles with good dispersion potential under aqueous conditions is one of the key concerns associated with practical applications of nanoparticles in medicine and industry. Researchers across the globe have been looking for appropriate green protocols for improving the dispersion stability of nanoparticles for wider application and performance [30]. During present investigations, we noted that the silver nanoparticles formed with mannitol as well as yeast extract were well dispersed and stable, and showed no signs of agglomeration even on storing for a period of over a month at room temperature (Figure 4b). Some of the biomolecules including polyols are known to act as stabilizing agents [31]–[33].

### Conclusions

In summary, our results demonstrated for the first time that (i) inclusion of Ag^+^ in YEM medium prior to autoclaving lead to generation of silver nanoparticles and therefore, it is not appropriate to include silver salt in the culture medium prior to autoclaving for evaluating the impact of Ag^+^ on microorganisms; (ii) YEM medium and two of its constituents namely, yeast extract and mannitol promote rapid generation of nanoparticles by autoclaving (as autoclaving promotes generation of silver nanoparticles under strict sterile conditions, the same can be used directly in medicine and pharmaceutical industry); and (iii) the silver nanoparticles generated on autoclaving AgNO_3_ with yeast extract and mannitol remain stable in aqueous phase under ambient conditions for long durations.
